# Supplementary material for: Causal Relationship between Aging and Anorexia Nervosa: A White-Matter-Microstructure-Mediated Mendelian Randomization Analysis
Source: Biomedicines. 2024 Aug 16;12(8):1874. doi: 10.3390/biomedicines12081874 (PMC11351342; doi:10.3390/biomedicines12081874)
Supplement: Supplementary file 1 [file biomedicines-12-01874-s001.zip › Supplementary Figure/Figure S4/Singe SNP plot of MR analysis for TL to ubm-b-1485.pdf]

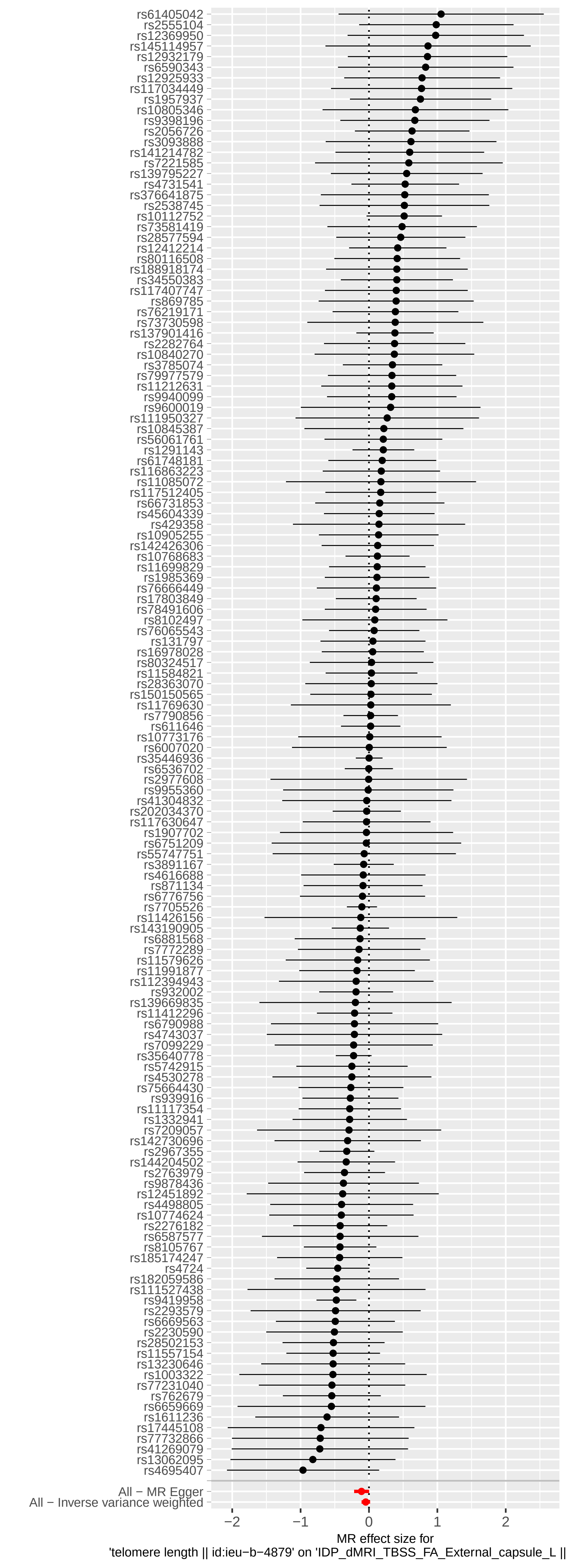

rs61405042
rs2555104
rs12369950
rs145114957
rs12932179
rs6590343
rs12925933
rs117034449
rs1957937
rs10805346
rs9398196
rs2056726
rs3093888
rs141214782
rs7221585
rs139795227
rs4731541
rs376641875
rs2538745
rs10112752
rs73581419
rs28577594
rs12412214
rs80116508
rs188918174
rs34550383
rs117407747
rs869785
rs76219171
rs73730598
rs137901416
rs2282764
rs10840270
rs3785074
rs79977579
rs11212631
rs9940099
rs9600019
rs111950327
rs10845387
rs56061761
rs1291143
rs61748181
rs116863223
rs11085072
rs117512405
rs66731853
rs45604339
rs429358
rs10905255
rs142426306
rs10768683
rs11699829
rs1985369
rs76666449
rs17803849
rs78491606
rs8102497
rs76065543
rs131797
rs16978028
rs80324517
rs11584821
rs28363070
rs150150565
rs11769630
rs7790856
rs611646
rs10773176
rs6007020
rs35446936
rs6536702
rs2977608
rs9955360
rs41304832
rs202034370
rs117630647
rs1907702
rs6751209
rs55747751
rs3891167
rs4616688
rs871134
rs6776756
rs7705526
rs11426156
rs143190905
rs6881568
rs7772289
rs11579626
rs11991877
rs112394943
rs932002
rs139669835
rs11412296
rs6790988
rs4743037
rs7099229
rs35640778
rs5742915
rs4530278
rs75664430
rs939916
rs11117354
rs1332941
rs7209057
rs142730696
rs2967355
rs144204502
rs2763979
rs9878436
rs12451892
rs4498805
rs10774624
rs2276182
rs6587577
rs8105767
rs185174247
rs4724
rs182059586
rs111527438
rs9419958
rs2293579
rs6669563
rs2230590
rs28502153
rs11557154
rs13230646
rs1003322
rs77231040
rs762679
rs6659669
rs1611236
rs17445108
rs77732866
rs41269079
rs13062095
rs4695407
All − MR Egger
All − Inverse variance weighted
−2
−1
0
1
2
MR effect size for
'telomere length || id:ieu−b−4879' on 'IDP_dMRI_TBSS_FA_External_capsule_L ||
